# Enhance Wine Production Potential by Using Fresh and Dried Red Grape and Blueberry Mixtures with Different Yeast Strains for Fermentation

**DOI:** 10.3390/foods12213925

**Published:** 2023-10-26

**Authors:** Juan Martín-Gómez, Teresa García-Martínez, M. Ángeles Varo, Julieta Mérida, María P. Serratosa

**Affiliations:** 1 Department of Agricultural Chemistry, Soil Science and Microbiology, Campus of Rabanales, Universidad de Córdoba, Bd, Marie Curie, E-14014 Córdoba, Spain; mi2gamam@uco.es (T.G.-M.); q72vasam@uco.es (M.Á.V.); jmerida@uco.es (J.M.); mpserratosa@uco.es (M.P.S.); 2 Institute of Chemistry for Energy and the Environment, Campus of Rabanales, Universidad de Córdoba, E-14014 Córdoba, Spain

**Keywords:** antioxidant activity, chamber-drying technique, grape–blueberry synergy, bioactive compounds, wines

## Abstract

Red grapes and blueberries are known for their high content of bioactive compounds and antioxidant properties. In Mediterranean winemaking, traditional sun-drying can be replaced by controlled-airflow-chamber-drying, which provides better quality, higher phenolic content, and increased antioxidants. This study aimed to increase the sugar content and phenolic compounds of the must by drying the fruits to fifty per cent of their original moisture content. Two musts were prepared: the first one was prepared by combining fresh red grapes and dried blueberries (*M*_1_), while the other was created using dried red grapes and fresh blueberries (*M*_2_), followed by fermentation at 25 °C with M05 Mead and X5 yeast strains. The M_2_ must showed the highest levels of phenolic compounds, red color (A520), total anthocyanins, and antioxidant activity. During fermentation, the anthocyanin content increased mainly in the dried blueberry macerates, where it increased between 4- to 5.5-fold. More bioactive compounds were extracted from the wines produced using yeast inoculation despite the shorter maceration times. A sensory analysis demonstrated consumers’ acceptance of the wines in terms of color, flavor, and aroma. In conclusion, the use of red grapes in the production of blueberry red wine proved to be effective, providing higher sugar and must yields, while the dried fruits improved the fermentable sugar content obtaining wines with an alcoholic content between 10 and 11% (*v*/*v*). The higher levels of bioactive compounds increased the antioxidant capacity of the resulting red fruit wines.

## 1. Introduction

Red grapes and blueberries are known for their high content of bioactive compounds and antioxidant activity due to their skin or pulp color [1,2,3]. These characteristics give them, among other fruits [4,5,6], numerous beneficial health properties, such as their anticancer, anti-inflammatory, and antimicrobial capacity. The presence of antioxidants in these fruits is particularly important as they can react with reactive oxygen species (ROS), which are unstable and highly reactive molecules present in the human body. When oxidized, antioxidant compounds help reduce oxidative stress, an imbalance that occurs when ROS exceed the body’s ability to neutralize them. Oxidative stress has been linked to premature aging and the development of various disorders, such as cardiovascular problems, neurodegenerative diseases, and some types of cancer. However, fresh blueberries and red grapes present a challenge in terms of preservation, as they are highly susceptible to mechanical damage and microbial degradation. This leads to a short shelf life and unavoidable economic losses. Since these fruits have a seasonal availability and a limited storage period, derivative products that preserve the bioactive compounds present in these fruits have been developed, turning them into functional foods. In the case of blueberries, several products have been created to make the most of these fruits and reduce waste. These products include juice, wine, vinegar, jam, dehydrated berries, powder pulp, dyes, and flavor additives used in the production of cakes, cookies, bread, yogurt, and jelly [7].

Zhu et al. [8] carried out a study with 234 consumers to investigate consumer preferences for fruit wines. The results revealed that grape wine and blueberry wine were the most favored by the study’s participants. However, wine-making blueberries present a problem due to their low sugar content, so, after fermentation, these beverages present an alcohol content of approximately 5–6% *v*/*v* [9,10]. According to the official definition of the International Organization of Vine and Wine (OIV), these products could not be called wine, since the latter’s alcoholic content cannot be less than 8.5% *v*/*v*. The most common method to compensate for this sugar deficiency is the direct addition of sucrose to the blueberry juice [11,12,13]. In a study by Liu et al. [14], the results revealed that additional sucrose prolongs the total fermentation time and increases the acidity of the wine. In addition, the color of the wine is affected, as the added sugar darkens and yellows the final product. Interestingly, sucrose has a protective effect on the anthocyanin levels, the compounds responsible for the color in blueberries.

Another way to address this problem and increase the sugar content of these beverages is through the post-harvest dehydration process of blueberries. This process has been shown to have significant impacts on the composition and characteristics of blueberries and their resulting wines. First, post-harvest dehydration has shown a decrease in the titratable acidity of both the blueberries and the wines made from them. In addition, moderate dehydration has been observed to increase the levels of anthocyanins and phenols in both the blueberries and the wines, which contribute to a higher content of health-promoting compounds [15]. Drying, from an oenological point of view, is an important process since many wines are made from dehydrated grapes [16]. In Mediterranean areas such as in the Montilla-Moriles appellation in southern Spain, the sun-drying of grapes is still used for the production of its sweet white wines. This type of drying usually extends over a range between five and ten days depending on the weather conditions each year. In addition, it presents a series of disadvantages as a consequence of working in the open air, such as occasional rains, high solar radiation, insect and animal attacks, or microbial attacks by toxin-producing fungi, such as ochratoxin A, among others [16,17,18,19]. This type of drying can be replaced by drying with air flow in a temperature-controlled chamber, which would avoid all these types of inconveniences. Some authors have shown that the use of this type of drying allows one to obtain higher-quality dried products than sun-dried ones and have found that the phenolic content and antioxidant activity of the berries are increased, in addition to the increase in the sugar content [17,20,21,22]. In a previous study, the vinification of sugar-enriched blueberry juice was investigated by pre-concentrating it through dehydration in a temperature-controlled drying chamber, achieving an alcoholic strength of 17% *v*/*v* [21].

Therefore, the aim of this work was to investigate the vinification of musts obtained from the combination of dried grapes or blueberries with fresh fruits, with the purpose that the dehydration of one of the fruits in the mixture would increase the sugar content and, consequently, the alcohol content of the resulting beverages. In addition, changes in the phenolic content, antioxidant activity, and acceptance of the wines produced by regular consumers were evaluated.

## 2. Materials and Methods

### 2.1. Materials

Grapes (*Vitis vinifera*), of the Tempranillo variety, were provided by the Instituto Andaluz de Investigación y Formación Agraria, Pesquera, Alimentaria y de la Producción Ecológica (IFAPA), in Cabra, Spain. Blueberries (*Vaccinium corymbosum*), of the Ventura variety, provided by the company PlusBerries, were harvested in the province of Huelva, Spain. Both fruits were harvested at their optimum ripeness for consumption (23 °Brix and 13 °Brix for grapes and blueberries, respectively), and were frozen at −18 °C until the time of analysis. Subsequently, to begin the experiments, they were thawed at 25 °C for 24 h.

### 2.2. Dehydration Process

The starting grapes and blueberries were dehydrated in a Frisol Climatronic drying chamber, with a relative humidity of 20% and an air current at a constant temperature of 50 °C. The drying process was controlled through the loss of fruit weight, with periodic weighing, and was maintained until both fruits lost 50% of their initial moisture. After the drying process, the fruits were blended using the same amount of undried fruit (2.5 kg) and dried fruit (2.5 Kg), thus obtaining two different musts (*M*_1_ and *M*_2_): one must was obtained from the fresh grapes and the dried blueberries (*M*_1_), with a total soluble solids (TSS) of 21.2 °Brix; the other must was obtained from the dried grapes and the fresh blueberries (*M*_2_), with a TSS of 18 °Brix.

To calculate the dry matter content of both fruits, they were dried in an oven at 100 °C until they reached a constant weight.

The moisture ratio (*MR*) was calculated using the following equation:

(1) $$MR=\frac{M_{t}-M_{e}}{M_{0}-M_{e}}$$

where *M_t_*, *M*_0_, and *M_e_* are the moisture content at a given drying time (kg water/kg dm), the initial moisture content (kg water/kg dm), and the equilibrium moisture content (kg water/kg dm), respectively. As the equilibrium moisture content is small relative to the others, it can be assumed that it is equal to zero [22], simplifying the equation to the following:

(2) $$MR=\frac{M_{t}}{M_{0}}$$

### 2.3. Selection and Preparation of Yeast Inocula

Two *Saccharomyces cerevisiae* yeast strains were selected for must fermentation: X5 (CECT131015) yeast isolated from partially sun-dried Pedro Ximenez grape musts [23], which are resistant to high concentrations of sugar and alcohol, and the commercial strain M05 Mead from Mangrove Jack’s (M), normally used in mead fermentation.

The preparation of the pre-inocula of the different strains was carried out in a YPD culture medium (1% *w*/*v* yeast extract, 2% peptone, 2% *w*/*v* D-glucose) at 28 °C and 150 rpm on the New Brunswick Scientific orbital shaker (Edison, NJ, USA) for 24 h.

### 2.4. Fermentation Process

For the development of the fermentation process, the musts previously mentioned in Section 2.2 (*M*_1_ and *M*_2_) were used. After blending the fruits, they were pressed together in a manual vertical press, performing two pressing cycles, with a maximum pressure of 300 bar. A total of 100 mL of the resulting musts together with 50 g of solid parts from the pressing were inoculated with the selected yeasts, with a cell concentration of 5 × 106 cells/mL, and introduced into thermostatized baths at 25 ± 0.2 °C. The monitoring of the fermentations was carried out using the weight difference, with the periodic weighing and the estimation of the CO_2_ released, since, according to reaction 1, it is stoichiometric with the production of ethanol in the medium.

C_6_H_12_O_6_ → 2 C_2_H_5_OH + 2 CO_2_ (3)

An aliquot of each must (*M*_1_ and *M*_2_) was used as a control and allowed to ferment spontaneously with the indigenous yeasts of the fruits used. The fermentation process was maintained until the alcoholic strength estimated using the sugar–alcohol correlation tables was reached, with 12.5% *v*/*v* of ethanol estimated for the *M*_1_ must and 10.6% *v*/*v* for the *M*_2_ must. To control the evolution of the fermentation, periodic weightings were carried out, which allowed us to approximate the alcohol content of our wines at each moment, related to the loss of CO_2_. Once the expected alcoholic content of the fermentations was reached, the yeast residues were removed from the medium using centrifugation and filtration.

The fermentations of each must (*M*_1_ and *M*_2_) under the three conditions (control, M05 Mead yeast, and X5 yeast) were carried out in duplicate (a and b), obtaining the wines shown in Table 1.

### 2.5. Alcohol Content

The procedure followed was the one proposed by Crowell and Ough, based on the steam entrainment of the ethanol contained in the sample and the subsequent reaction, at a controlled temperature, with a solution of potassium dichromate in an acid medium, performing a spectrophotometric measurement at 600 nm using a Beckman DU 640 UV-visible spectrophotometer and comparing the absorbance with that obtained in a standard line of ethanol [24].

### 2.6. Volatile Acidity

The isolation of volatile acids was carried out according to the OIV method by entrainment with water vapor and the rectification of the vapors [25]. The sample was acidified before the entrainment, taking care to ensure that it was free of carbon dioxide gas. The acidity was determined on the distillate, titrating with NaOH and using phenolphthalein as an indicator.

### 2.7. Spectrophotometric Determinations

Using a UV-visible spectrophotometer, Beckman DU 640, and quartz cuvettes with an optical pitch of 1 mm, the absorbance of the musts was measured at 420, 520, and 620 nm to estimate the contribution to the color of the brown, red, and blue compounds, respectively. In addition, the hue parameter was calculated with Formula (2) to determine the existence of a greater contribution from the brown or red compounds. All the absorbances were performed in triplicate for each independent fermentation and were corrected to an optical step of 1 cm.

(4) $$\mathrm{Hue}=\frac{\mathrm{Abs}\;420\;\mathrm{nm}}{\mathrm{Abs}\;520\;\mathrm{nm}}$$

### 2.8. Total Phenolic Compounds

The Folin–Ciocalteu method [26] was used for the determination of the total phenolic compounds in triplicate for each independent fermentation. For this, to 1.25 mL of Folin–Ciocalteu’s reagent diluted 1:5 with distilled water, 50 µL of the sample was added, shaken vigorously, and allowed to stand for 1 min. Then, 1 mL of 10% *w*/*v* sodium carbonate was added and left in the dark for 30 min. After this time, the blue coloration produced by the Folin–Ciocalteu reagent oxides related to the concentration of the phenolic compounds was measured at 760 nm using a Beckman DU 640 spectrophotometer. A calibration curve for the gallic acid was performed using different concentrations of standard in a range between 0.01 and 1 g gallic acid/L.

### 2.9. Total Flavonoids

For the determination of the total flavonoids in triplicate for each independent fermentation, the aluminum trichloride method was used. For this, 300 µL of the sample filtered through 0.45 µm was taken in each case, to which 120 µL of 2% (*w*/*v*) AlCl_3_ was added, and the volume was made up to 3 mL with 5% acetic acid in methanol. It was then allowed to stand in the dark for 30 min, and, finally, the absorbance was measured at 425 nm with a Beckman DU 640 UV-vis spectrophotometer. The data were expressed as mg quercetin/L. For this purpose, a quercetin calibration line between 0 and 700 mg quercetin/L was made.

### 2.10. Total Anthocyanins

To determine the total anthocyanin content in triplicate in each independent fermentation, the differential pH method was used [27]. For this, two 1:10 dilutions of the sample were prepared in two different buffers: KCl at pH 1 and NaCH_3_COO at pH 4.5. After a resting period of 20 min, the absorbance was measured using a Beckman DU 640 UV-vis spectrophotometer at 520 nm and 700 nm. The total anthocyanin content was calculated using Formulas (3) and (4), taking into account the following parameters: Mw (molecular weight of cyanidin-3-glucoside, 449.2 g/mol), D (dilution factor), ε (molar absorptivity of cyanidin-3-glucoside, 26,900 L/mol-cm), and PL (optical light path).

(5) $$\mathrm{Total}\;\mathrm{anthocyanins}\;(\mathrm{mg}/L)=\frac{A\times M_{w}\times D\times 1000}{\varepsilon \times PL}$$

A = (A_520_ − A_700_)_pH1_ − (A_520_ − A_700_)_pH4,5_ (6)

### 2.11. Antioxidant Activity

#### 2.11.1. DPPH Assay

The DPPH assay was used to determine the antioxidant activity of the musts and the wines that had been obtained in triplicate for each independent fermentation, according to the method used by Katalinic et al. [28], with some modifications. To 3 mL of the DPPH 45 mg/L solution, 200 µL of the sample diluted 1:10, or 200 µL of water in the case of the control, was added. The absorbance of the control was measured immediately at 517 nm using a Beckman DU 640 spectrophotometer, while the sample was measured after 30 min of incubation at room temperature and in darkness. A calibration curve was performed with Trolox in the range of 10–200 mg Trolox/L, and the percentage inhibition was calculated according to Formula (5).

(7) $$\mathrm{Percentage}\;\mathrm{inhibition}=\frac{{\mathrm{Abs}}_{\mathrm{control}}-{\mathrm{Abs}}_{\mathrm{sample}}}{{\mathrm{Abs}}_{\mathrm{control}}}\;\text{×}\;100$$

#### 2.11.2. ABTS Assay

The ABTS assay was developed according to the method proposed by Re et al. [29], with some modifications. The ABTS^+^ radical was formed by oxidation of 7 mM of ABTS solution with 2.45 mM of potassium persulfate; the mixture was kept in the dark for 12 h to complete the reaction. Subsequently, the ABTS^·+^ radical solution was diluted with ethanol until the absorbance at 734 nm reached a value of 0.700 ± 0.020. Next, 900 µL of this diluted solution was taken and 100 µL of the sample, or 100 µL of distilled water in the case of the control, was added. The absorbance of the control was measured immediately at 734 nm using a Beckman DU 640 spectrophotometer, while that of the sample was measured after 6 min at room temperature and in darkness. The percentage inhibition was calculated with Formula (6), and the antioxidant activity was established with the help of a calibration line of Trolox in the range 10–100 mg Trolox/L.

(8) $$\mathrm{Percentage}\;\mathrm{inhibition}=\frac{{\mathrm{Abs}}_{\mathrm{control}}-{\mathrm{Abs}}_{\mathrm{muestra}}}{{\mathrm{Abs}}_{\mathrm{control}}}\;\text{×}\;100$$

### 2.12. Sensory Analysis

In the sensory analysis, a tasting card was prepared to evaluate the color, flavor, and aroma parameters of the different wines obtained by three expert tasters (winery oenologists) and twenty regular consumers in two panels in accordance with ISO 8586:2023 [30] The tasting room was kept at 20 °C and the musts were served in tasting glasses certified in accordance with UNE 87022:1992 [31]—the Spanish equivalent of ISO 3591:1977 [32]. The glasses were coded with tree-digit blinding codes and covered to prevent sensory losses in the musts. The tasters were instructed in advance about their task and the rules to be followed and were given a scoring sheet.

The evaluation of the quality of the musts was made using the method according to ISO 4121:2003 [33]. The different parameters were evaluated with the following options: desirable (5–6), acceptable (3–4) and undesirable (1–2).

- First panel: evaluation of aroma and flavor.
- Second panel: evaluation of color.

### 2.13. Statistical Analysis

The results of all the samples were subjected to an analysis of variance at a 99.0% confidence level. Homogeneous groups were calculated to establish significant differences between means. A simple linear correlation was made between the antioxidant activity values and the total phenolic compounds and anthocyanins content. The data obtained from the drying processes were adjusted to different mathematical models frequently used to model drying curves. The software used was Statgraphics Centurion XVI.

## 3. Results and Discussion

**Figure 1:**
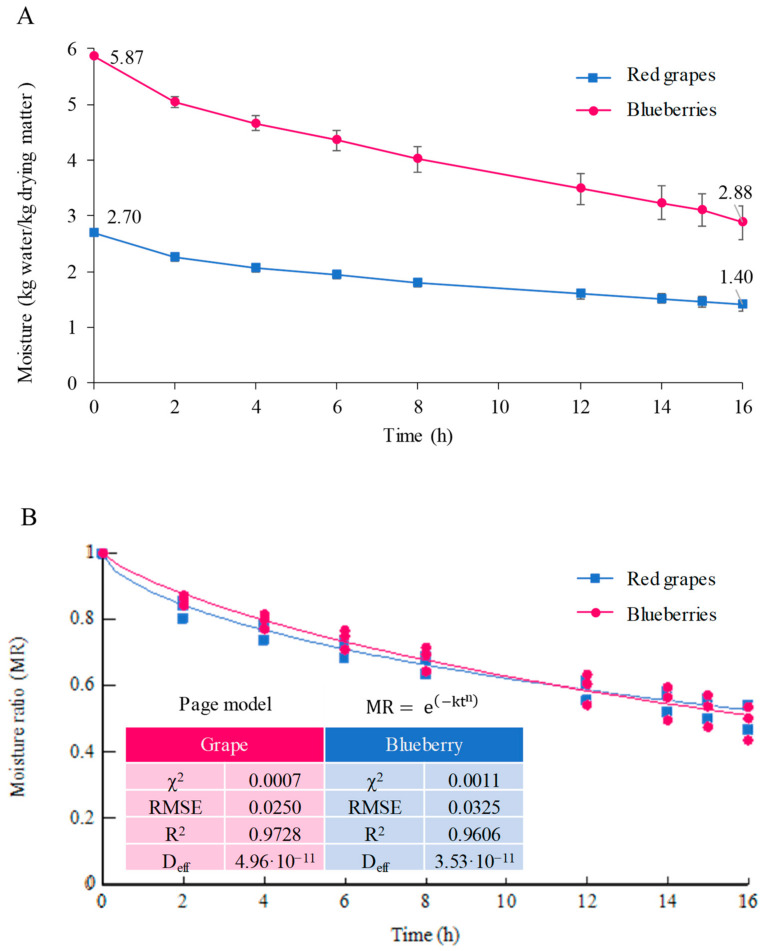
Dying curves of the blueberries and the red grapes: moisture vs. time (**A**) and moisture ratio vs. time (**B**).

The dehydration process of the red grapes and the blueberries was developed to increase the sugar and phenolic compound contents in the musts to be fermented. The temperature chosen for both drying processes was 50 °C, based on what was reported by other authors [22,34] who, studying the drying of these fruits, had concluded that 50 °C was the temperature at which the dehydration process was faster, maintaining a greater amount of phenolic compounds and antioxidant activity. On the one hand, the drying process was monitored by measuring the moisture loss and expressing it in kg water/kg drying matter (Figure 1). As can be seen, the blueberries initially contained a greater initial amount of water than the grapes (5.87 vs. 2.70 kg w/Kg dm). Considering that both dryings were maintained for 16 h until the fruits lost 50% of their initial moisture, this implies that the dried blueberries were left with a higher moisture content than the dried grapes (2.88 vs. 1.40 Kg w/Kg dm). On the other hand, it is important to consider, in the drying processes, the variation of the moisture ratio, determined as the ratio of the moisture content at each time-point to the initial moisture content versus the drying time (Figure 1).

As can be seen from the figure, the moisture ratio decreased exponentially over time, in both processes, in the same way, indicating that the processes were carried out at the same rate. Specifically, considering that the optimal criteria to evaluate the best fit and the quality of the fit to the mathematical models is to have the highest R^2^ value and the lowest χ^2^ and RMSE values, different mathematical models were compared to study the fit of the drying curves (approximation of diffusion, Page, and two-term models). The best fit in both processes was the “Page model”, with the R^2^ values of 0.9728 and 0.9606 for the blueberries and the red grapes drying, respectively, in addition to possessing the lowest χ^2^ and RMSE values, indicating that, with this model, the changes in the fruit moisture content with the drying time could be predicted. This was the same model that had been employed to previously record the blueberries’ drying-rate at that temperature [34].

**Figure 2:**
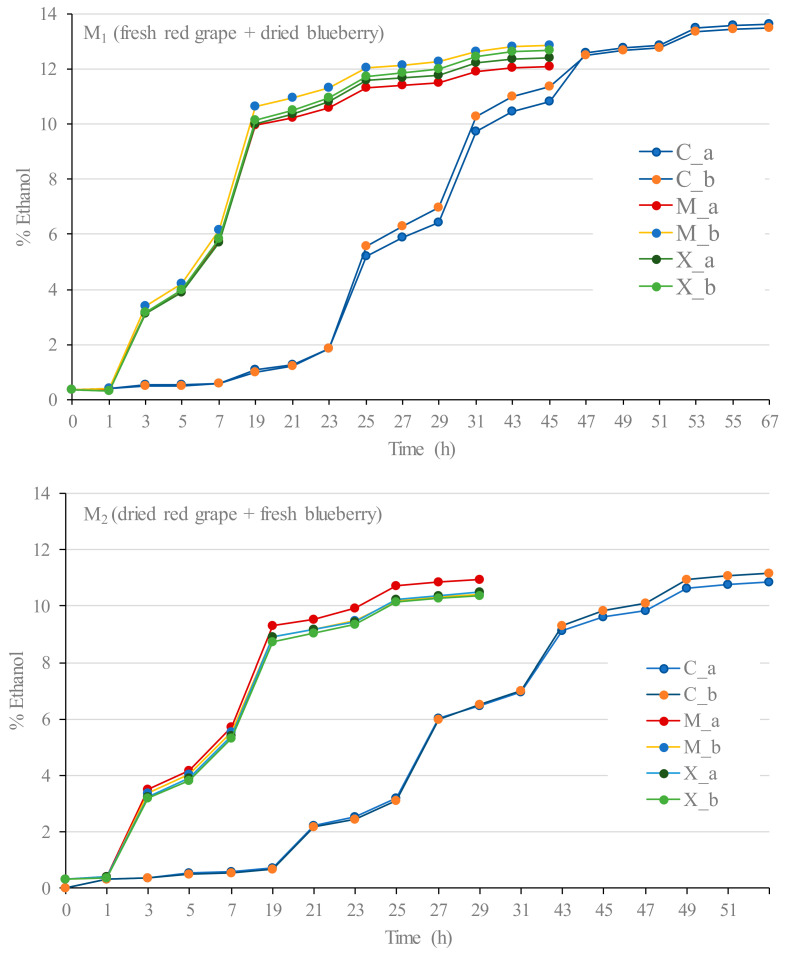
Fermentation curves of the fresh red grapes and dried blueberries must (*M*_1_) and the dried red grapes and fresh blueberries must (*M*_2_) for the following: a control (C_a and C_b); with M05 Mead yeast (M_a and M_b); and X5 yeast (X_a and X_b).

Figure 2 shows the fermentation progress of the musts *M*_1_ (fresh red grapes + dried blueberries) and *M*_2_ (dried red grapes + fresh blueberries), inoculated with the selected yeasts (M05 Mead and X5), and the fermentation of a control must (not inoculated), which was left to ferment spontaneously with the indigenous yeasts present in the fruits.

The difference in the fermentation times of the musts was caused by the different initial TSS. The fermentations carried out with the M_2_ must were the first to finish (29 h) because they consumed all the sugar content: these included both of the musts inoculated with the M05 Mead and X5 yeasts, with a final alcohol content of 10.3 and 10.5% *v*/*v*, respectively (Table 2). Then, after 45 h, the four inoculated fermentations of M_1_ musts were finished, with a final alcohol content between 10.8 and 11.6% *v*/*v*. Finally, the control fermentations carried out without an inoculum ended after 51 h in the case of those from the M_2_ must, with an alcohol content of approximately 9.5% *v*/*v*, and after 67 h in the case of those from the M_1_ must, with an alcohol content of approximately 11.4% *v*/*v*.

The wines produced in this work do not exceed the allowed volatile acidity limit of 7.9 meq/L for grape wines [35] since they all presented lower values (Table 1). However, it can be seen that the control wines produced using spontaneous fermentation showed higher volatile acidity values than the wines produced after inoculation. This may be due to the fact that, in the control musts, the fermentation process was longer, meaning that a greater number of secondary reactions could occur. In addition, the wines made with the X5 yeast (W_1_X and W_2_X) had the lowest volatile acidity values, which could mean that this type of yeast causes the sugars to mainly go via the metabolic route for the production of alcohol and not participate in secondary reactions, such as the formation of acetic acid, or do so to a lesser extent.

The color of a fruit is the result of a complex mixture of pigments, which do not remain the same qualitatively or quantitatively but change over time, due to chemical reactions that occur between the pigments and the oxygen or other compounds present in the wine [36]. In wine, color is an important quality parameter, since it can be one of the first attributes that define it organoleptically. Table 3 shows the values of the absorbances at 420, 520, and 620 nm and the color intensity and hue of the initial musts and of the different wines obtained. Comparing the two starting musts, the must obtained from the mixture of dry grapes and fresh blueberries (*M*_2_) had a higher absorbance at 520 nm than the M_1_ must (2.30 vs. 1.68 a.u., respectively). This result confirms what has been reported in previous works [22,34], that is the fact that fresh blueberries contribute more red compounds than fresh grapes, namely 0.495 versus 0.087 a.u., respectively.

The absorbance at 420 nm is related to the coloration provided by brown compounds, and previous studies have shown that fresh grapes provide a lower concentration of these compounds than fresh blueberries [22,34]. The *M*_1_ must made with dried blueberries showed a higher value of A420 than when the blueberry was fresh, indicating that, during the drying process, the brown compounds increased due to the concentration effect of water evaporation and enzymatic and non-enzymatic browning reactions.

The hue is a ratio between the contribution of brown compounds versus red compounds, so values above one would indicate a higher contribution of brown compounds. In the case of young red wines, this value is in the range of 0.5–0.7 and increases with aging to a range of 1.2–1.3 [37]. The great difference in tonality between the two starting musts can be observed, with the value of the *M*_1_ must being the only one above one, with a value almost double that of the *M*_2must_ (1.10 vs. 0.642, respectively). This is a consequence of the higher value of A420 and the lower value of 520 in the *M*_1must_.

**Figure 3:**
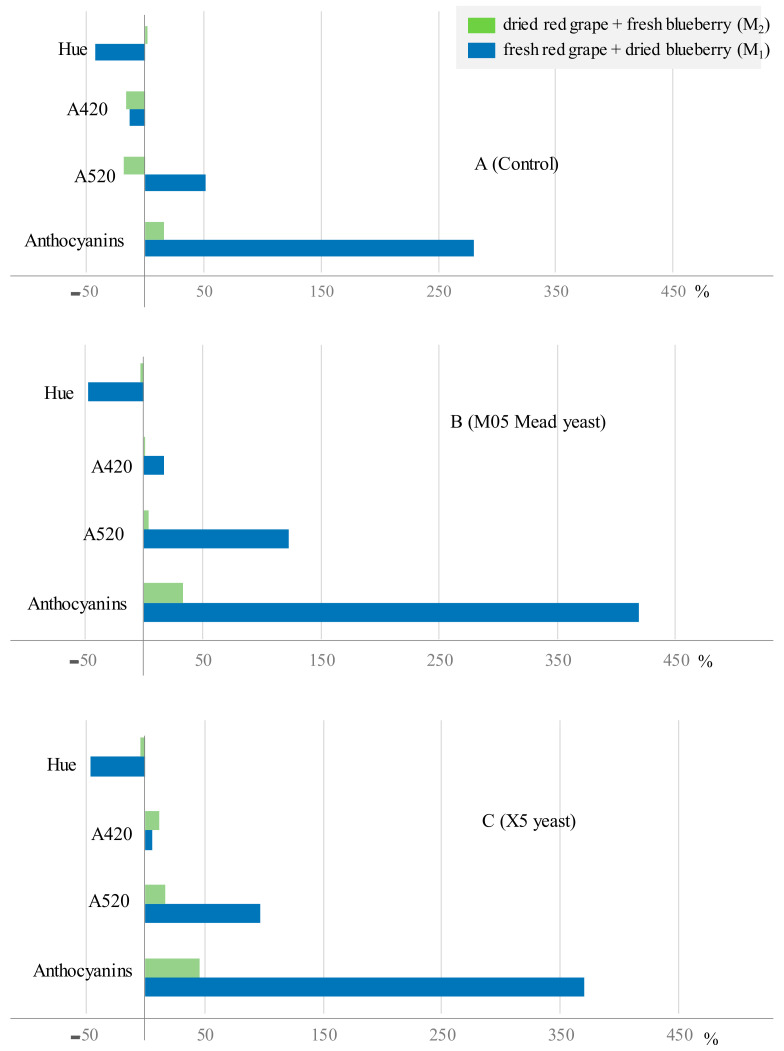
Percentage of change of anthocyanin content and color parameters in the control fermentations (**A**) and with the two selected yeasts (**B**,**C**).

After the fermentation of the *M*_1must_, an increase in the absorbance at 520 nm was observed in all the wines (Table 3). The W_1_M wines presented the highest absorbance values and the W_1_C control the smallest values, indicating that, when the maceration was carried out using dried blueberries, the extraction of red pigments was much higher because of the fact that, in that case, for the same weight of dried fruit, the proportion of skins in the blueberries was higher. Figure 3 shows the variation of A520 from the initial musts to the final wines. It can be seen that this variation occurred in both the control wine and in those that had been inoculated with yeast. Consequently, when the must was macerated using fresh blueberries (*M*_2_), a loss of color could be observed compared to the starting must (W_2_C) and a slight increase in the other two wines was obtained (W_2_M and W_2_X). This marked increase in A520 in the vinification of the *M*_1_ must caused a considerable decrease in hue, and the corresponding wines were found to have a hue between 0.578 and 0.646. However, during the fermentation of the M_2_ must, the hue was maintained, meaning that the finished W_2_ wines showed hues similar to those of the W_1_. Table 1 shows the anthocyanin content of the initial musts and of all the wines obtained after fermentation. It can be seen that, as mentioned for the A520, the *M*_2must_ obtained from the mixture of dry grapes and fresh blueberries presented a concentration that was slightly over three times that of the *M*_1must_ (9.08 vs. 2.91 mg/L), indicating that the red color mentioned was due to the anthocyanin derivatives, confirming that fresh blueberries have a higher concentration of these compounds than fresh grapes [22,34]. Figure 3 shows the variation in anthocyanin content from the initial musts to the final wines. It can be seen that, in the three fermentations carried out, the highest extraction occurred whilst obtaining Wine 1 (W_1_) as a consequence of the fact that, since the maceration was carried out using the same weight of fruit, the dry blueberries they were macerated with a much higher proportion of skins. This justifies extracting 280 vs. 16.5% in the control wines, 419 vs. 32.8% in the wines obtained with M05 Mead, and 370 vs. 45.3% in the wines obtained with X05.

The Folin–Ciocalteu index is a widely used method to measure the total phenolic compound content of wines, although other compounds could be included to a lesser extent. Table 1 shows the values of the polyphenol index of the initial musts and of the different wines obtained.

First of all, it can be seen that the musts coming from the pressing of a mixture of dried grapes and fresh blueberries (*M*_2_) presented a higher phenolic content than those coming from a mixture of fresh red grapes and dried blueberries (*M*_1_), i.e., 988 vs. 845 mg/L, as mentioned above, as a consequence of their much higher content of red pigments. In addition, it can be seen that these contents increased during vinification of the M_1_ must, due to the extraction of these compounds through the presence of the fruit skins during fermentation. Of these, the wines obtained by inoculation with M05 Mead yeast (W_1_M_a W_1_M_b) had the highest total phenolic content, followed by those inoculated with X5 (W_1_X_a W_1_X_b) and, finally, the control wines (W_1_C_a and W_1_C_b), with values of 917 and 912 mg/L, respectively, which, despite being the wines that underwent the longest fermentation process, i.e., the longest maceration, did not acquire a higher content.

In the vinification of the *M*_2_ must, as in the previous case, the wines fermented with pre-inoculum showed higher contents of total phenolic compounds. The capacity of yeasts to adsorb phenolic compounds during fermentation at the same time as they are extracted from the fruit skins is a well-known fact. It is also known that, at the end of fermentation, the yeasts experience a process of autolysis and a subsequent settling to the bottom of the vessel, carrying away the coloring and bioactive compounds that had remained adhered to the cell walls of the yeasts [38]. Therefore, it could be concluded that, although the control fermentations also had the presence of yeast, their duration was much longer, so that, probably, after a certain fermentation time, the extraction rate was less than their adsorption rate by the yeast [38].

All the wines obtained after the fermentation process of the mixed blueberry and grape musts (W_1_ and W_2_) presented a content of total phenolic compounds within the range, i.e., 912–1068 mg gallic acid/L, a range of values that are similar to those studied in blueberry wine by other authors such as Su and Chien [10], whose findings showed a content of total phenolic compounds in the range of 858–1150 mg gallic acid/L, or those studied by authors Jonhson and Gonzalez de Mejia [39], whose findings were in the range of 966–2510 mg gallic acid/L. Even the wines produced in this study showed a higher phenolic content than the blueberry wines produced by the authors Jonhson et al. [40], with a phenolic content between 375 and 657 mg gallic acid/L, or those elaborated by Zhang et al. [13] using ten different blueberry varieties, with a content in the range of 506–888 mg/L, with the exception of the Gardenblue variety, which had a content of 1205 mg gallic acid/L.

In relation to the flavonoid content, once again, the musts from the pressing of a mixture of dried red grapes and fresh blueberries (*M*_2_) showed a higher content than those from a mixture of fresh grapes and dried blueberries (*M*_1_), i.e., 30.6 vs. 27.9 mg/L, respectively. During vinification, the contents increased in all the wines, although the increases in the W_1_ wines were greater, with the result that the W_1_ wines had a higher flavonoid concentration.

Berries such as red grapes and blueberries have a high antioxidant activity, which gives them important beneficial properties for one’s health [41,42]. Antioxidant activity was measured with two methods whose names are given to the colored molecule used as a proton or electron scavenger. The DPPH assay reaction consists of an electron transfer followed or preceded by a proton transfer, known as a coupled proton–electron transfer reaction [43], whereas the ABTS reaction consists only of an electron transfer [29]. Therefore, the results obtained using both methods cannot be directly compared, since they present different reaction mechanisms [44].

Firstly, it can be seen that the musts from the pressing of a mixture of dried red grapes and fresh blueberries (*M*_2_) showed a higher antioxidant activity than those from a mixture of fresh red grapes and dried blueberries (*M*_1_), both for the DPPH assay (532 vs. 432 mg Trolox/L) and for the ABTS assay (1106 vs. 824 mg Trolox/L).

**Figure 4:**
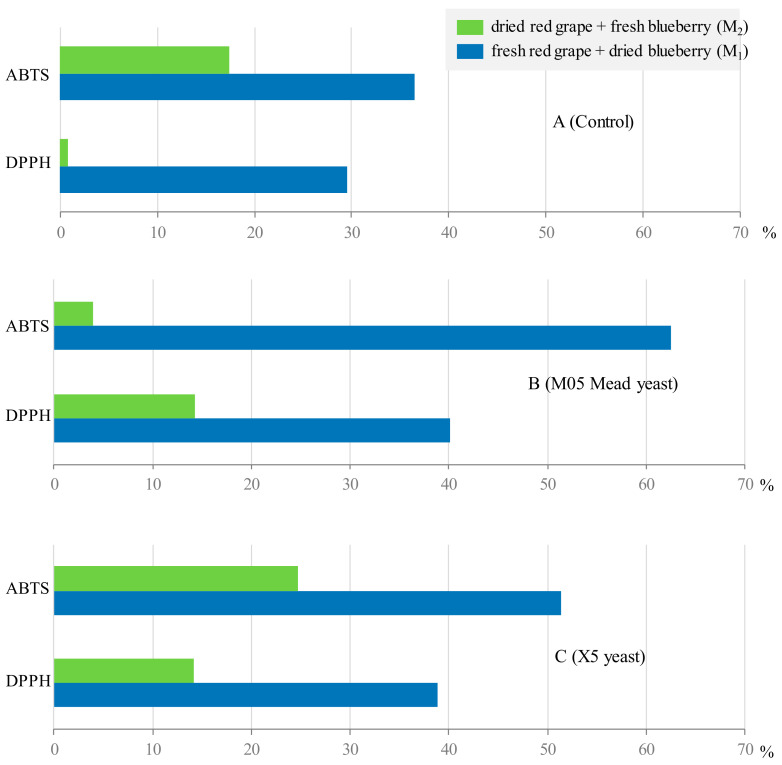
Percentage variation of antioxidant activity values in the control fermentations (**A**) and with the two selected yeasts (**B**,**C**).

It should be highlighted that, during the fermentation of the *M*_1_ must, as it can be seen in Figure 4, there is a large increase in antioxidant activity for both the DPPH and ABTS assays, leading to higher values in the ABTS assay. For this assay, the wines obtained via fermentation with the M05 Mead yeast showed the highest antioxidant capacity, registering an increase of more than 60% (62.5%), followed by the W_1_X wines (increase of 51.4%), and, finally, the W_1_C control wines, with an increase of 36.5% with respect to the starting must, which showed the lowest value.

**Figure 5:**
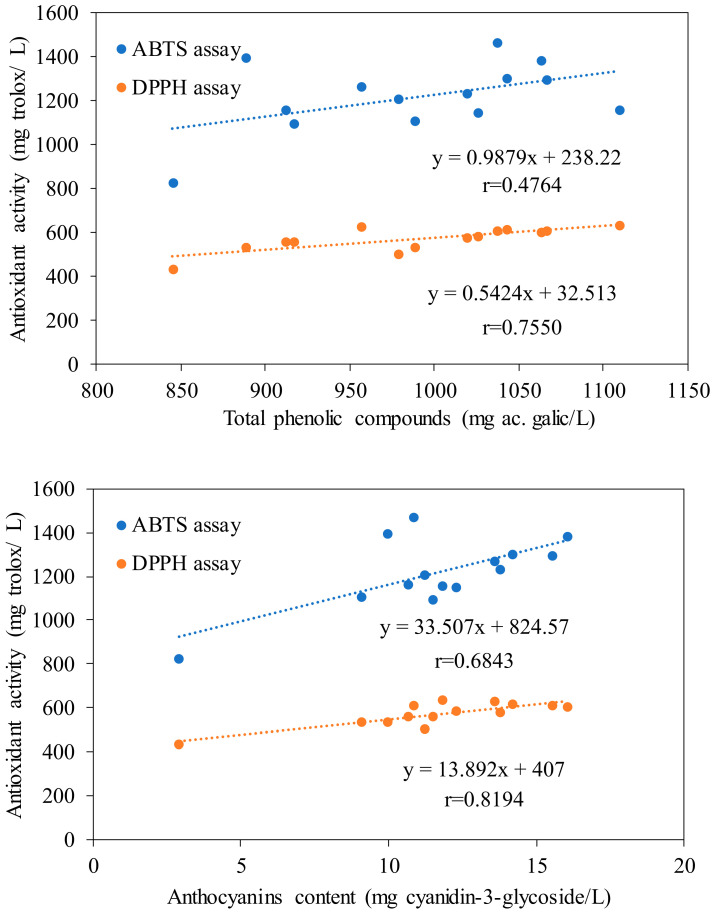
Correlations between antioxidant activity and total polyphenol and anthocyanin content.

In order to determine the relationship between antioxidant activity and phenolic compounds, a simple linear regression adjustment was carried out, measuring Pearson’s r correlation coefficient both for the ABTS assay and for the DPPH assay (Figure 5). Likewise, and due to its great influence on these fruits, a correlation study was carried out between the antioxidant activity and the total anthocyanin content (Figure 5). For the twelve degrees of freedom of the analysis (number of samples −2), the linear correlation coefficient has a significance of 95% (*p* < 0.05) when r ≥ 0.532, of 99% (*p* < 0,01) when r ≥ 0.661, and of 99.9% (*p* < 0.001) when r ≥ 0.780. As it can be seen, the variation of antioxidant activity is related to the total anthocyanin content, both when determined using the ABTS assay (*p* < 0.01) and when determined through the DPPH assay, even at an even higher significance level (*p* < 0.001). This supports what has been reported in the literature regarding the fact that these phenolic compounds are highly valued for their antioxidant properties, both as free radical scavengers and metal chelators, which are the reason for their benefits to human health. When the correlation is made with the content of phenolic compounds, it can be seen that this only presented a significance higher than 95% when performed with the DPPH assay (*p* < 0.01). It seems reasonable to think that the ABTS radical may have an affinity for other families of non-phenolic compounds, hence its lower significance in the correlation with antioxidant activity [29,43,44].

**Figure 6:**
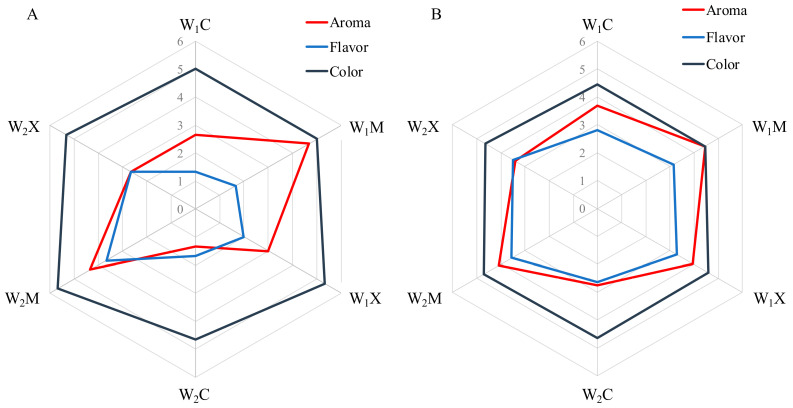
Scores obtained for the wines by the panel of expert tasters (**A**) and by the panel of regular consumers (**B**).

Figure 6 shows the results of the sensory analysis carried out, in which the organoleptic quality parameters such as color, aroma, and flavor were evaluated using the following evaluation criteria and scores: undesirable (1–2), acceptable (3–4), and desirable (5–6). Figure 6A shows the scores given by the three expert tasters, who concluded that, in aroma, the best wine was the one made from the fresh grapes and the dried blueberries (M_1_) and inoculated with M05 Mead yeast (W_1_M). They also found that the W_1_M wine had notes of ripe fruit, strawberry, and banana and that the W_2_M wine had notes of ripe fruit, blueberry jam, licorice, and banana. In relation to the wines made with the X5 yeast, the experts found that the W_1_X5 wine gave notes of dried fruit skin, herbal, menthol, and toasted aromas and that the W_2_X5 wine had notes of ripe fruit, peach, passion fruit, and licorice. Finally, the worst scores were awarded to the spontaneously fermented wines, although to a lesser extent for the W_1_C wine. In these wines, the only aroma notes that could be appreciated were those of ethyl acetate, due to the amount of acetic acid present. In flavor, the scores were not very high due to the acidity of the wines in which an excess of malic acid had been sensorially detected, with the exception of the wines W_2_M and W_2_X5, which were within the acceptable range. On the other hand, in color, all the wines scored very similarly within the desirable range due to their violet and burgundy hues characteristic of both fruits.

Figure 6B shows the data and scores obtained by the twenty tasters, who were regular wine consumers. As with the expert tasters, the wines made with pre-inoculums had the highest scores. In all the wines, color was the best evaluated parameter, with scores that classified it as acceptable. On the other hand, the best evaluated aroma was that of the wines that had been inoculated with the M05 Mead yeast (W_1_M and W_2_M), which classified them as acceptable. In terms of flavor, all the wines showed very similar scores in the acceptable range. It should be taken into account that the wines produced are new products that do not exist in the market, making the tasting more complex for regular red wine consumers. Perhaps the most interesting thing is that in no case were the wines rejected for any of their attributes.

## 4. Conclusions

Despite the fact that the blueberries initially contained a greater amount of water than the grapes, both dried fruits experienced the same kinetics according to the same regression model (Page model), taking the same time to reach 50% of their initial moisture content.

The must obtained from the mixture of dried red grapes and fresh blueberries had the highest content of phenolic compounds, A520, total anthocyanins, and antioxidant activity measured using both the ABTS and DPPH assays. On the other hand, the must obtained with fresh red grapes and dried blueberries had the highest content in reduced sugar, flavonoids, A420, and tonality. Therefore, it could be concluded that the use of blueberries favored the higher content of bioactive compounds in the musts, while grapes were the ones to provide the highest amount of sugars. During fermentation, the concentration of anthocyanins increased in all the wines obtained, with those macerated using dried blueberries multiplying their concentration by between 4 and 5.5 times, meaning that the drying of this fruit is the one that contributed the reddest color to the wine. The wines obtained using yeast inoculation extracted the most bioactive compounds, even though their maceration time was shorter. However, a longer fermentation time may instead cause the extracted phenolic compounds to be adsorbed by the yeasts during the autolysis process.

The sensory analyses carried out on the wines obtained show that, in all the cases, they were accepted by the consumers in terms of color, flavor, and aroma, with the wines obtained using inoculation with the M05 Mead being the most highly valued.

Therefore, the use of red grapes to produce blueberry red wine was appropriate because they provided sugars and higher yields of must. In addition, the use of dried fruit increased the content of fermentable sugars to a greater extent, and the maceration with the solid parts, particularly of the dried blueberries, increased the content of bioactive compounds and, consequently, the antioxidant capacity of the red fruit wines obtained.

## Figures and Tables

**Table 1:** Nomenclature used to designate the different wines produced.

	W_1_		W_2_	
	a	b	a	b
Control	W_1_C_a	W_1_C_b	W_2_C_a	W_2_C_b
M05 Mead yeast	W_1_M_a	W_1_M_b	W_2_M_a	W_2_M_b
X5 yeast	W_1_X_a	W_1_X_b	W_2_X_a	W_2_X_b

**Table 2:** Enological parameters of the initial musts and all the elaborated wines studied (*n* = three, mean ± standard deviation).

	Ethanol	Volatile Acidity	Total Phenolic Compounds	Total Flavonoids	Total Anthocyanins	Antioxidant Activity	
						DPPH Assay	ABTS Assay
*M*_1_	0	0	845 ± 2.28	27.9 ± 2.62	2.91 ± 0.094	432 ± 2.72	824 ± 43.1
W_1_C_a	11.4 ± 0.012	7.64 ± 0.246	917 ± 0.228	37.5 ± 0.131	11.5 ± 0.155	560 ± 13.3	1093 ± 3.29
W_1_C_b	11.3 ± 0.067	6.17 ± 0.246	912 ± 4.53	39.4 ± 1.19	10.6 ± 0.106	561 ± 28.0	1157 ± 15.2
W_1_M_a	10.5 ± 0.056	3.73 ± 0.248	1043 ± 7.09	41.1 ± 0.758	14.2 ± 0.149	612 ± 8.52	1300 ± 15.9
W_1_M_b	11.6 ± 0.281	3.95 ± 0.000	1063 ± 4.48	47.9 ± 0.845	16.0 ± 0.291	600 ± 3.84	1380 ± 2.86
W_1_X_a	10.8 ± 0.080	1.74 ± 0.248	957 ± 2.00	40.3 ± 1.22	13.6 ± 0.235	626 ± 2.84	1264 ± 25.0
W_1_X_b	10.8 ± 0.080	1.74 ± 0.248	1019 ± 4.33	40.4 ± 0.675	13.8 ± 0.072	575 ± 15.5	1231 ± 23.7
*M*_2_	0	0	988 ± 3.22	24.1 ± 0.611	9.08 ± 0.384	532 ± 0.620	1106 ± 9.61
W_2_C_a	9.6 ± 0.059	7.15 ± 0.246	988 ± 1.70	30.6 ± 0.007	9.96 ± 0.181	533 ± 1.43	1393 ± 13.4
W_2_C_b	9.4 ± 0.065	7.40 ± 0.000	979 ± 4.74	33.1 ± 0.801	11.2 ± 0.073	540 ± 0.562	1205 ± 10.9
W_2_M_a	10.5 ± 0.106	2.73 ± 0.248	1109 ± 6.48	36.4 ± 0.420	11.8 ± 0.127	634 ± 4.20	1155 ± 23.2
W_2_M_b	10.3 ± 0.049	2.96 ± 0.000	1025 ± 51.8	33.1 ± 0.210	12.3 ± 0.068	583 ± 0.580	1146 ± 11.2
W_2_X_a	10.4 ± 0.039	1.74 ± 0.248	1037 ± 5.96	34.9 ± 0.250	10.8 ± 0.162	606 ± 0.926	1466 ± 40.5
W_2_X_b	10.5 ± 0.112	1.99 ± 0.000	1066 ± 9.15	36.5 ± 1.23	15.5 ± 1.40	610 ± 9.38	1294 ± 9.30

**Table 3:** Color parameters of the initial musts and all the elaborated wines studied (*n* = three, mean ± standard deviation).

	A420	A520	A620	Color Intensity	Hue
M_1_	1.85 ± 0.010	1.68 ± 0.003	0.410 ± 0.003	3.94 ± 0.009	1.10 ± 0.004
W_1_C_a	1.59 ± 0.005	2.58 ± 0.000	0.358 ± 0.003	4.52 ± 0.002	0.615 ± 0.002
W_1_C_b	1.61 ± 0.001	2.50 ± 0.005	0.345 ± 0.005	4.45 ± 0.001	0.646 ± 0.001
W_1_M_a	2.11 ± 0.008	3.66 ± 0.023	0.497 ± 0.004	6.26 ± 0.034	0.578 ± 0.001
W_1_M_b	2.23 ± 0.029	3.83 ± 0.043	0.526 ± 0.020	6.59 ± 0.092	0.583 ± 0.001
W_1_X_a	2.05 ± 0.004	3.47 ± 0.011	0.511 ± 0.001	6.03 ± 0.015	0.592 ± 0.001
W_1_X_b	1.84 ± 0.004	3.14 ± 0.006	0.436 ± 0.002	5.41 ± 0.008	0.586 ± 0.000
M_2_	1.47 ± 0.007	2.30 ± 0.034	0.400 ± 0.003	4.17 ± 0.044	0.642 ± 0.006
W_2_C_a	1.23 ± 0.004	1.84 ± 0.019	0.272 ± 0.001	3.34 ± 0.024	0.667 ± 0.005
W_2_C_b	1.24 ± 0.006	1.94 ± 0.021	0.253 ± 0.002	3.43 ± 0.029	0.641 ± 0.004
W_2_M_a	1.50 ± 0.007	2.32 ± 0.015	0.316 ± 0.004	4.13 ± 0.025	0.643 ± 0.001
W_2_M_b	1.49 ± 0.002	2.44 ± 0.011	0.343 ± 0.002	4.27 ± 0.015	0.607 ± 0.002
W_2_X_a	1.65 ± 0.005	2.64 ± 0.017	0.385 ± 0.001	4.67 ± 0.023	0.622 ± 0.002
W_2_X_b	1.65 ± 0.001	2.70 ± 0.007	0.419 ± 0.001	4.76 ± 0.005	0.611 ± 0.002

## Data Availability

Not applicable.
